# Plasma Macrophage Migration Inhibitory Factor Predicts Graft Function Following Kidney Transplantation: A Prospective Cohort Study

**DOI:** 10.3389/fmed.2021.708316

**Published:** 2021-09-01

**Authors:** Yongrong Ye, Fei Han, Maolin Ma, Qipeng Sun, Zhengyu Huang, Haofeng Zheng, Zhe Yang, Zihuan Luo, Tao Liao, Heng Li, Liangqing Hong, Ning Na, Qiquan Sun

**Affiliations:** ^1^Division of Kidney Transplantation, Organ Transplantation Research Institution, The Third Affiliated Hospital, Sun Yat-sen University, Guangzhou, China; ^2^Department of Kidney Transplantation, Guangdong Provincial People's Hospital, Guangzhou, China

**Keywords:** kidney transplantation, delayed graft function, macrophage migration inhibitory factor, biomarker, graft function

## Abstract

**Background:** Delayed graft function (DGF) is a common complication after kidney transplantation (KT) with a poor clinical outcome. There are no accurate biomarkers for the early prediction of DGF. Macrophage migration inhibitory factor (MIF) release during surgery plays a key role in protecting the kidney, and may be a potential biomarker for predicting post-transplant renal allograft recovery.

**Methods:** Recipients who underwent KT between July 2020 and December 2020 were enrolled in the study. Plasma MIF levels were tested in recipients at different time points, and the correlation between plasma MIF and DGF in recipients was evaluated. This study was registered in the Chinese Clinical Trial Registry (ChiCTR2000035596).

**Results:** Intraoperative MIF levels were different between immediate, slowed, and delayed graft function groups (7.26 vs. 6.49 and 5.59, *P* < 0.001). Plasma MIF was an independent protective factor of DGF (odds ratio = 0.447, 95% confidence interval [CI] 0.264–0.754, *P* = 0.003). Combining plasma MIF level and donor terminal serum creatinine provided the best predictive power for DGF (0.872; 95%CI 0.795–0.949). Furthermore, plasma MIF was significantly associated with allograft function at 1-month post-transplant (*R*^2^ = 0.42, *P* < 0.001).

**Conclusion:** Intraoperative MIF, as an independent protective factor for DGF, has excellent diagnostic performance for predicting DGF and is worthy of further exploration.

## Introduction

Kidney transplantation (KT) is the primary therapy for end-stage renal disease (ESRD), with a lower mortality rate and superior quality of life compared to dialysis (1, 2). However, early complications limit the benefit of KT, such as delayed graft function (DGF), which is often defined as requiring dialysis within 1 week following KT (3, 4). The rate of DGF in recipients is as high as 20–33% (5), and the strategy of using an extended criteria donor to extend the donor pools significantly increases the rate of DGF (6–8). DGF increases the costs of hospitalization, prolongs the length of stay, increases the rate of acute rejection, and affects the renal allograft prognosis (9–11). There are no accurate biomarkers for the early prediction of DGF, and there remains an urgent need for relevant indications.

Macrophage migration inhibitory factor (MIF) is a pleiotropic cytokine that is rapidly released following various stimuli from cellular stores, compared with other cytokines that require mRNA transcription and protein synthesis before release (12, 13). MIF is a 12.5-kDa homotrimer that binds and activates various receptors (13, 14). Previous studies have shown that MIF has antioxidant properties (15–17), and its binding to CD74 exerts a protective role in cardiac ischaemia reperfusion injury (IRI) by antioxidant effects through the CD74/CD44/AMP-activated protein kinase pathway (18, 19). A retrospective study also reported that elevated MIF levels were negatively correlated with cardiac dysfunction after a heart operation (20). In addition, the rate of acute kidney injury (AKI) was dramatically reduced in cardiac surgery patients with high circulating MIF at 12 h postoperatively (21). Similar to heart operations, transplant surgery with blockade of blood flow is associated with tissue hypoxia and systemic oxidative stress, leading to elevated MIF in circulation (22). However, the relationship between elevated MIF during KT and post-transplant renal allograft function has not been studied. We hypothesized that elevated circulatory MIF during KT alleviates IRI of the graft, thereby reducing the rate of DGF and improving postoperative renal allograft function.

This study explored the correlation between plasma MIF levels and postoperative renal allograft function in recipients. It may provide transplant physicians with an early, non-invasive, and accurate means of predicting DGF.

## Methods

### Study Design

We conducted a prospective and observational cohort study. This study enrolled 85 recipients who received allografts from patients declared brain death between July 2020 and December 2020 at the Third Affiliated Hospital of Sun Yat-sen University. Exclusion criteria for recipients were as follows: (1) without blood samples or with haemolysis of blood samples; (2) patients who were administered different doses of glucocorticoids (GCs) during surgery due to GC-induced MIF release (23); (3) patients undergoing simultaneous multiple organ transplantation. All patients were followed up for 1 month after KT.

The study was approved by the ethics committee of the Third Affiliated Hospital of Sun Yat-sen University, and it complied with the Declarations of Helsinki and Istanbul (24). All enrolled recipients agreed to participate in this study and signed an informed consent form. The China Organ Transplant Response System completed organ allocation according to equitable and transparent principles (25). This study was registered in the Chinese Clinical Trial Registry (ChiCTR2000035596).

### Data Source and Immunosuppressive Regimen

Donor data were acquired from the organ procurement organizations. Clinical transplant surgeons provided recipient data.

We divided the recipients into three groups: immediate graft function (IGF), slowed graft function (SGF), and delayed graft function (DGF). The creatinine reduction ratio (CRR) was used to diagnose IGF and SGF. The definition of DGF was requiring dialysis within 1 week following KT. SGF was defined as CRR <70% and without the need for dialysis within 1 week after KT. IGF was defined as CRR>70% in the week after KT and without the need for dialysis (9).

Immunosuppressive induction regimens were performed by antithymocyte globulin (50 mg/day, 0–day 2) or basiliximab (20 mg/day, 0 and day 4). Methylprednisolone was usually administered intravenously at a dose of 500 mg/day during inducing therapy and administration of the first dose was completed before kidney reperfusion. Calcineurin inhibitor, mycophenolate mofetil and prednisone were combined to maintain immunosuppression. The onset doses of oral tacrolimus or cyclosporine were 0.1–0.15 or 6–8 mg/kg/day on day 2–4, respectively. The doses were adjusted according to required blood concentrations. Mycophenolate mofetil was started post-transplant and was maintained at a dose of 1.5–2 g/day. Oral prednisone was administered at 30 mg/day after inducing therapy and was decreased by 5 mg/week to a maintenance daily dose of 10–15 mg.

### Sample Collection and Detection

Blood samples were acquired from the first 30 recipients at the following timepoints: after anesthesia and before skin incision (pre-operation), 5 min after kidney reperfusion (during operation), and the end of surgery (post-operation). The last 47 recipient blood samples were only collected once 5 min after kidney reperfusion (during operation). Samples were placed in an ice box and immediately sent to the laboratory to prepare the plasma. The blood samples were immediately centrifuged at 1,500 rpm/min for 10 min, and the supernatants were transferred to the cryotubes with a pipette, and then stored at the −80°C until analysis.

We used an enzyme-linked immunosorbent assay (Human MIF ELISA Kit; ABclonal Technology, Wuhan, China) to measure plasma MIF concentrations according to the manufacturer's protocol. This assay employs the quantitative sandwich enzyme immunoassay technique wherein the MIF-specific monoclonal antibody is pre-coated on the microplate, and any MIF present in the standard and sample is bound by the immobilized antibody. The MIF-specific detection antibody binds to the combination of the capture antibody-MIF in the sample. Adding enzyme conjugate and substrate successively leads to the formation of a colored product TMB. The reaction was terminated by adding acid, and the absorbance is measured. The intensity of the TMB was proportional to the amount of MIF present in the sample. Finally, the concentration of the MIF sample was determined by plotting a curve of the absorbance for the sample and standard.

### Statistical Analysis

The Mann-Whitney *U*-test was used to compare the differences between different groups for nonnormally distributed variables, and Student's *t*-test was used to test whether there is a difference between different groups on a continuous dependent variable. The parametric analysis of variance (ANOVA) and Kruskal-Wallis tests were used to analyse the differences in biomarker levels between different groups. The diagnostic performance of MIF and other biomarkers for the prediction of DGF was evaluated by receiver operating characteristic (ROC) curve analyses. The independent relevant parameters of DGF were acquired with multivariate logistic regression analyses using a backward stepwise method, and the method of stepwise analysis was set as *P* < 0.05 for entry and *P* > 0.1 for removal. These independent relevant parameters obtained from the multivariate logistic regression analyses were used to build a predictive model for DGF. The optimal cut-off points for the ROC curves analyses were calculated using Youden's J-statistic. Spearman's correlation coefficient was used to analyse the correlation between MIF and recipient serum creatinine (Scr). The correlation between MIF levels and Scr levels at 1-month post-transplant was evaluated by the multiple linear regression analysis. Statistical analysis was carried out using SPSS version 25.0.

## Results

### Demographic Data of Recipients and Donors

**Figure 1:**
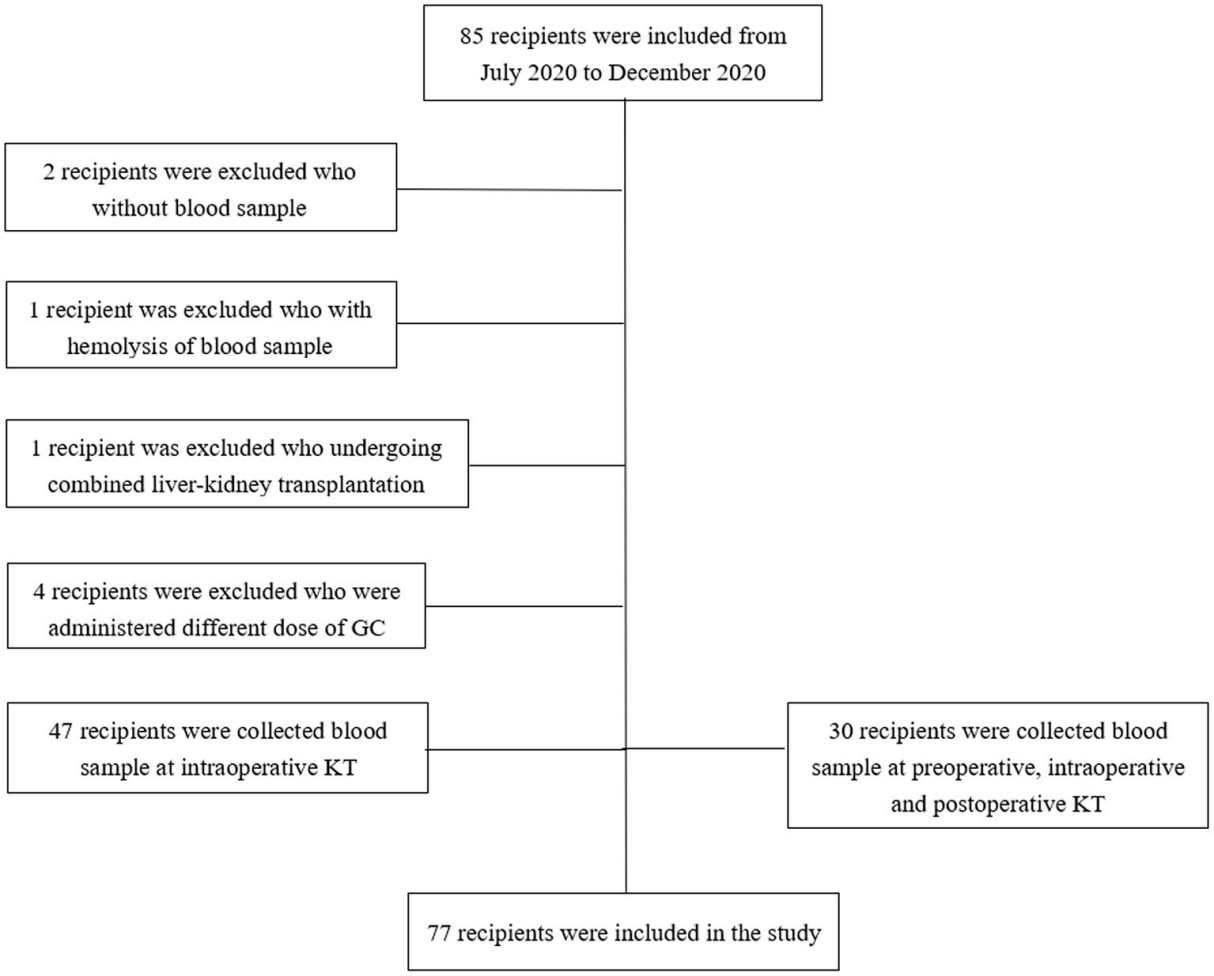
Flow chart of kidney recipients enrolled in the study.

Of the 85 recipients enrolled, 8 recipients were excluded: 2 without plasma samples, 1 with haemolysis of plasma sample, 4 were administered different doses of GCs during surgery, and 1 had undergone combined liver-kidney transplantation (Figure 1). Demographic data of the 77 recipients and their donors are presented in Table 1.

**Table 1:** Summary of characteristics in recipient and donor, stratified by recipients' s allograft function.

**Characteristic**		**All (*n* = 77)**	**IGF (*n* = 39)**	**SGF (*n* = 16)**	**DGF (*n* = 22)**	***P-*value**
**Recipients**						
Age, years		42 [34.5,51]	43 [35,51]	40 [36,51.5]	45.5 [31.8,54]	0.029
Male sex		46 (60)	22 (56)	7 (44)	17 (77)	0.096
Cause of ESRD						0.63
	GN	16 (21)	8 (21)	2 (13)	6 (27)	
	Hypertension	2 (3)	0 (0)	1 (6)	1 (5)	
	Diabetes	2 (3)	1 (3)	0 (0)	1 (5)	
	Others	57 (74)	30 (77)	13 (81)	14 (64)	
Mode of dialysis						0.628
	HD	66 (86)	33 (85)	15 (94)	18 (82)	
	PD	6 (8)	3 (8)	0	3 (14)	
	WD	5 (6)	3 (8)	1 (6)	1 (4)	
Dialysis duration, mo		12 [3.5,22.5]	12 [4,25]	5.5 [1.1,59.8]	12 [4.8,30]	0.713
Cold ischaemic time, h		4 [3,5.5]	3.5 [3,4.5]	5 [4,6]	5 [4,6]	<0.001
Number of HLA mismatches		5 [4,5]	5 [4,5]	5 [4.3,5.8]	5 [4,5]	0.362
Panel reactive antibody						0.358
	0%	66 (86)	34 (87)	12 (75)	20 (91)	
	1–10%	11 (14)	5 (13)	4 (25)	2 (9)	
Induction regimen						0.381
	ATG	51 (66)	28 (72)	11 (69)	12 (55)	
	Basiliximab	26 (34)	11 (18)	5 (31)	10 (45)	
CNI						0.368
	Cyclosporin	2 (3)	2 (5)	0 (0)	0 (0)	
	Tacrolimus	75 (97)	37 (95)	16 (100)	22 (100)	
Steroid		77	39	16	22	
Renal functions at post-transplant (mg/dL)						
	Scr, at 1 day	9.13 [6.34,11.22]	8 [5.15,11.11]	9.2 [6.11,10.24]	10.68 [7.9,11.84]	0.064
	Scr, at 1 week	2.87 [1.62,5.46]	1.63 [1.17,2.14]	4.32 [3.28,6.65]	6.32 [4.71,9.36]	<0.001
	Scr, at 1 month	1.75 [1.46,2.28]	1.5 [1.23,1.82]	1.92 [1.66,2.27]	2.71 [1.86,4.21]	<0.001
Variation of Scr after transplantation (mg/dL)						
Absolute decrease 0 h−1 day		0.94 [−0.76,3.32]	3.04 [0.94,4.62]	−0.49 [−1.19,0.76]	−0.03 [−1.22,1.38]	<0.001
Relative decrease (0 h−1 day)/1 day		0.12 [−0.08,0.34]	0.28 [0.11,0.41]	−0.05 [−0.17,0.1]	0 [−0.11,0.14]	<0.001
MIF levels during operation (ng/mL)		6.52 [5.59,7.45]	7.26 [6.28,8.39]	6.49 [5.52,7.21]	5.59 [4.36,6.03]	<0.001
**Donors**						
Age, years		51 [38.5,54]	42 [38,54]	52 [41,55]	51.5 [46,54.3]	0.207
Male sex		52 (68)	23 (59)	12 (75)	17 (77)	0.264
BMI (kg/m^2^)		23.4 [20.8,24.8]	22.9 [19.4,24.1]	23.8 [22.5,25.5]	24.1 [22.8,26.5]	0.017
Hypertension		43 (56)	19 (49)	10 (63)	14 (64)	0.442
Cause of death						0.526
	Head trauma	7 (9)	2 (5)	2 (13)	3 (14)	
	Stroke	51 (66)	25 (64)	12 (75)	14 (64)	
	Other	19 (25)	12 (31)	2 (13)	5 (23)	
Admission Scr, mg/dL		1.28 [0.85,2.1]	0.97 [0.7,1.28]	1.59 [1.1,2.43]	2.26 [1.43,3.34]	<0.001
Terminal Scr, mg/dL		1.46 [0.87,2.02]	1.01 [0.7,1.48]	1.64 [1.11,2.41]	1.94 [1.69,4.83]	<0.001
Use of any vasoactive drugs		71 (92)	35 (90)	15 (94)	21 (95)	0.703
No. of any vasoactive drugs		4 [2,8]	3 [2,6]	4.5 [2.8]	5.5 [2,9]	0.544
No. of kidneys transplanted	1	5 (6)	1 (3)	1 (6)	3 (14)	0.241
	2	72 (94)	38 (97)	15 (94)	19 (86)	

*According to Shapiro-Wilk test, if P > 0.05 for continuous variables, the data are presented as mean ±SD; otherwise, the data are presented as median [P25, P75]; categorical variables are expressed by total numbers and percentages*.

*ANOVA or Kruskal–Wallis tests were used to analyze continuous variables and χ^2^ test and Fisher's exact test were used to analyze categorical variables*.

*DGF, delayed graft function; ESRD, end-stage renal disease; GN, glomerulonephritis; HD, hemodialysis; PD, peritoneal dialysis; WD, without dialysis; HLA, human leukocyte antigen; ATG, Rabbit Anti-Human Thymocyte Immunoglobulin; CNI, calcineurin inhibitor; Scr, serum creatinine; MIF, Macrophage migration inhibitory factor; BMI, body mass index; AKI, acute kidney injury; ANOVA, analysis of variance*.

All recipients were divided into 3 groups according to allograft function: 39 had IGF, 16 had SGF, and 22 had DGF. Recipient age was different between the IGF, SGF, and DGF groups (43 years vs. 40 and 45.5 years, *P* = 0.029). The cold ischaemic time of the kidney in the IGF group was significantly lower than that in the DGF and SGF groups (3.5 h vs. 5 and 5 h, *P* < 0.001). Donor body mass index was significantly different between the IGF, SGF, and DGF groups (22.9 kg/m^2^ vs. 23.8 and 24.1 kg/m^2^, *P* = 0.017). There were no differences in the mode of dialysis, dialysis duration, cause of ESRD, induction regimen, number of HLA mismatches, panel reactive antibody, donor cause of death, use of vasoactive drugs, or number of vasoactive drugs used between the three groups (Table 1).

### Differences in Plasma MIF in Perioperative Patients Undergoing Kidney Transplant

The differences in plasma MIF between 30 patients with DGF or non-DGF during the perioperative period are shown in Supplementary Figure 1. There was no difference between DGF and non-DGF patients in baseline plasma MIF at pre-transplant timepoints (1.28 ng/mL vs. 1.17 ng/mL, *P* = 0.428). However, intraoperative and postoperative plasma MIF levels were significantly higher in the non-DGF group than in the DGF group, and the intraoperative difference was more evident between groups (7.02 ng/mL vs. 5.18 ng/mL, *P* < 0.001; 6.25 ng/mL vs. 5.5 ng/mL, *P* = 0.028, respectively). Plasma MIF levels significantly increased at intraoperative and postoperative timepoints compared to pre-operation (6.56 ng/mL vs. 0.71 ng/mL, *P* < 0.001; 5.86 ng/mL vs. 0.71 ng/mL, *P* < 0.001, respectively), but there was no difference between intraoperative and postoperative timepoints (Supplementary Figure 2). Therefore, we used the intraoperative period as the timepoint at which to detect MIF.

### Distinction of Intraoperative Plasma MIF Among 77 Patients of Different Graft Function

**Figure 2:**
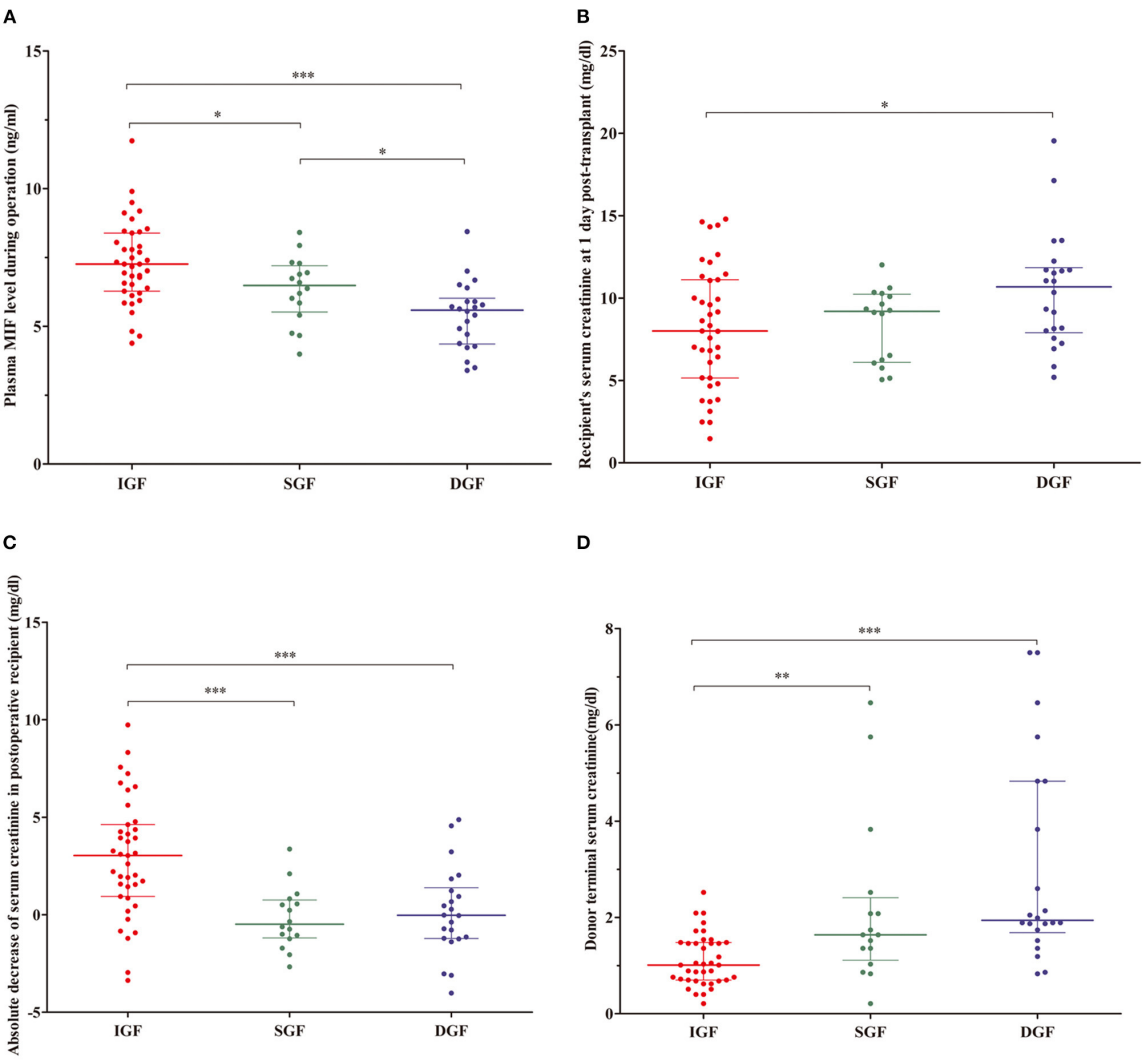
**(A)** Distributions of plasma macrophage migration inhibitory factor (MIF) from recipient during operation, recipient's serum creatinine (Scr) at 1-day post-transplant, absolute Scr decrease from 0-h to 1-day post-transplant, and donor terminal Scr in the recipient. **(B–D)** Contents of MIF, recipient' Scr at 1-day post-transplant, absolute Scr decrease after transplant, and donor terminal Scr in immediate graft function (IGF; red), slowed graft function (SGF; green), and delayed graft function (DGF; blue) groups. *P < 0.05, **P < 0.01, ***P < 0.001.

The intraoperative plasma MIF in the IGF group was higher than that in the SGF and DGF groups (7.26 ng/mL vs. 6.49 ng/mL, *P* < 0.05; 7.26 ng/mL vs. 5.59 ng/mL, *P* < 0.001, respectively), and the SGF group was also higher than that in the DGF group (6.49 ng/mL vs. 5.59 ng/mL, *P* < 0.05) (Figure 2A). Recipient Scr at 1-day post-transplant was different between the IGF and DGF groups (8 mg/dL vs. 10.68 mg/dL, *P* = 0.033; Figure 2B). A decrease in recipient Scr from 0-h to 1-day post-transplant and donor terminal Scr effectively distinguished the IGF group from the SGF and DGF groups (3.04 mg/dL vs. −0.49 and −0.03 mg/dL, *P* < 0.001; 1.01 mg/dL vs. 1.64 and 1.94 mg/dL, *P* < 0.001, respectively), but neither indicators differed between the SGF and DGF groups (Figures 2C,D). According to the intraoperative plasma MIF level distribution, the incidence of needing dialysis within 1 week after transplantation increased 6-fold when the levels were <5.8 ng/mL (67 vs. 11%, *P* < 0.001).

### MIF Is an Independent Protective Factor for Delayed Graft Function

The relevant parameters for predicting DGF were acquired using univariate and multivariate logistic regression analyses. From the univariate analyses, we concluded that recipient intraoperative plasma MIF, donor terminal Scr, and kidney cold ischaemia time were significantly correlated with DGF (odds ratio [OR] = 0.396, 95% confidence interval [CI] 0.242–0.649, *P* = 0.001; *OR* = 1.857, 95%CI 1.261–2.736, *P* = 0.002; *OR* = 1.552, 95%CI 1.097–2.195, *P* = 0.013). From multivariate logistic regression analyses, we further concluded that plasma MIF and donor terminal Scr were independent relevant parameters for DGF (*OR* = 0.447, 95%CI 0.264–0.754, *P* = 0.003; *OR* = 1.648, 95%CI 1.108–2.449, *P* = 0.014; Table 2).

**Table 2:** Univariate and multivariate logistic regression analyses for predicting delayed graft function.

	**Univariate**			**Multivariate**		
	**OR**	**95%CI**	***P*-value**	**OR**	**95%CI**	***P*-value**
Donor age (years)	1.03	0.989–1.072	0.157			
Donor sex (women)	0.515	0.165–1.607	0.253			
Donor BMI (kg/m^2^)	1.123	0.977–1.29	0.102			
Donor cause of death	0.747	0.306–1.821	0.521			
Donor terminal Scr (mg/dL)	1.857	1.261–2.736	0.002	1.648	1.108–2.449	0.014
Cold ischaemic time (h)	1.552	1.097–2.195	0.013			
Recipient age (years)	0.998	0.952–1.046	0.921			
Recipient sex (female)	0.328	0.106–1.014	0.053			
Duration of dialysis before transplantation (mo)	1.005	0.992–1.018	0.474			
HLA mismatch	0.897	0.524–1.537	0.693			
PRA	0.511	0.101–2.582	0.417			
Induction regimen	2.031	0.732–5.640	0.174			
Plasma MIF (ng/mL)	0.396	0.242–0.649	0.001	0.447	0.264–0.754	0.003

*Multivariate logistic regression analysis was executed with a backward selection procedure*.

*HLA, human leukocyte antigen; PRA, panel reactive antibody; MIF, Macrophage migration inhibitory factor; BMI, body mass index; AKI, acute kidney injury; Scr, serum creatinine; CI, confidence interval; OR, odds ratio*.

### Predictive Value of Plasma MIF for Delayed Graft Function

**Figure 3:**
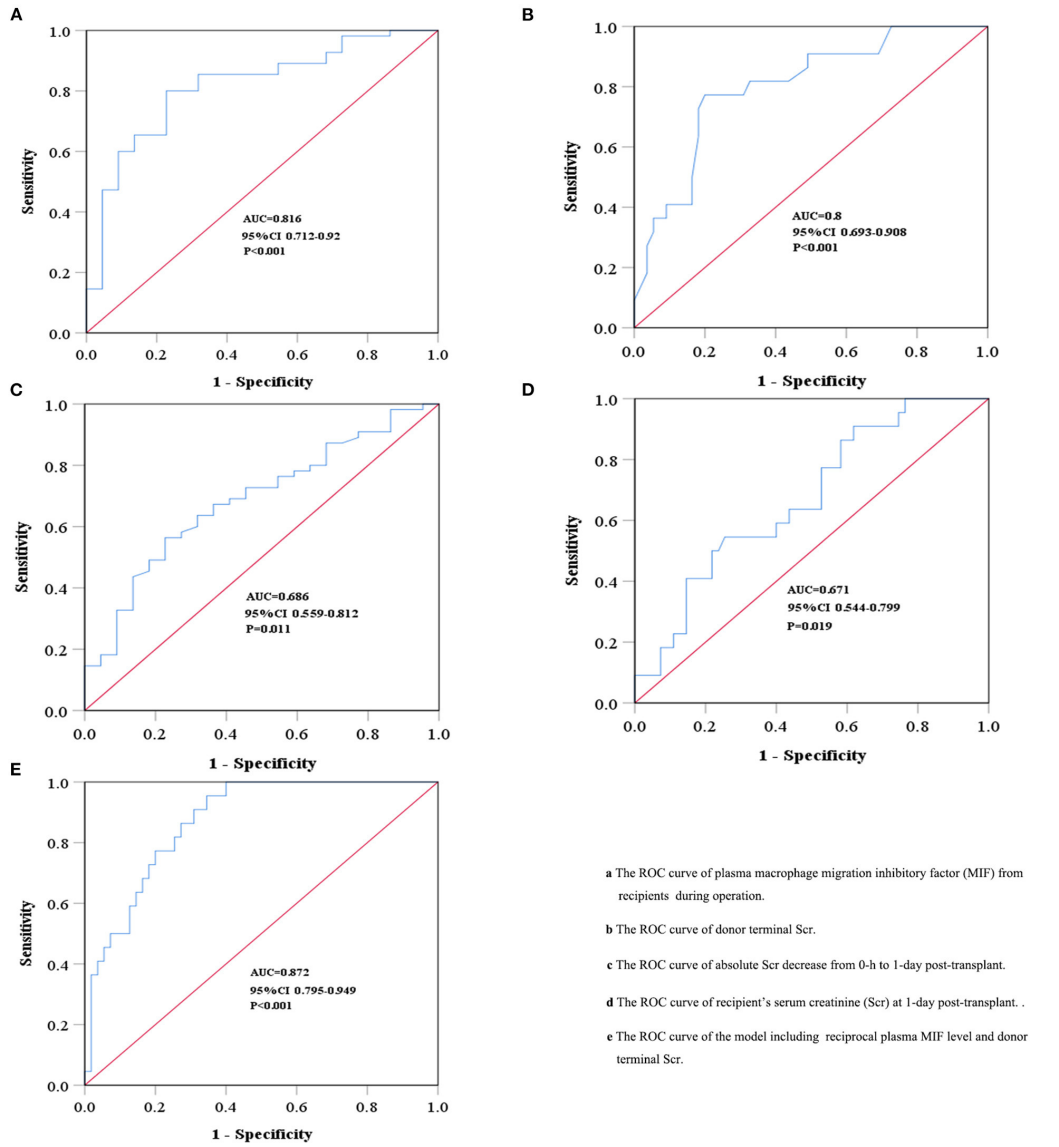
Receiver operating characteristic (ROC) curves for recipient and donor characteristics for predicting delayed graft function. The ROC curves of plasma macrophage migration inhibitory factor (MIF) from recipients during operation **(A)**, donor terminal Scr **(B)**, absolute Scr decrease from 0-h to 1-day post-transplant **(C)**, and recipient's serum creatinine (Scr) at 1-day post-transplant **(D)**. The linear prediction model including the reciprocal plasma MIF level and donor terminal Scr **(E)**.

We used ROC curve analysis to evaluate the diagnostic performance of plasma MIF, the recipient's Scr at 1-day post-transplant, absolute decreased Scr after transplant, and donor terminal Scr for predicting DGF. The area under the ROC curve (AUROC) of plasma MIF and donor terminal Scr in predicting DGF was 0.816 (95%CI 0.712–0.92, *P* < 0.001) and 0.8 (95%CI 0.693–0.908, *P* < 0.001), respectively, which were superior to that of the recipient's Scr at 1-day post-transplant (0.671; 95%CI 0.544–0.799, *P* = 0.019 or absolute decreased Scr after transplant (0.686; 95%CI 0.559–0.812, *P* = 0.011; Figures 3A–D). Meanwhile, we combined the relevant parameters that were acquired from multivariate logistic regression analyses to build a predictive model for DGF. The model including the reciprocal plasma MIF level and donor terminal Scr improved the AUROC to 0.872 (95%CI 0.795–0.949, *P* < 0.001; Figure 3E). Moreover, this model had higher sensitivity and moderate specificity at the optimum cut-off point (sensitivity = 0.96, specificity = 0.66). The sensitivity, specificity, and optimal cut-off point of these parameters for predicting DGF are shown in Table 3.

**Table 3:** Sensitivity, specificity, and predictive values for predicting delayed graft function at optimum cut-off value of recipient and donor characteristic.

	**Cut-off value**	**Sensitivity**	**Specificity**	**PPV**	**NPV**	**Youden index**	**AUC (95%CI)**
A—Recipient's Scr at 1-day post-transplant (mg/dL)	6.88	0.91	0.38	0.37	0.91	0.29	0.671 (0.544–0.799)
B—Plasma MIF level (ng/mL)	5.92	0.8	0.77	0.58	0.91	0.57	0.816 (0.712–0.92)
C—Donor terminal Scr (mg/dL)	1.73	0.77	0.80	0.61	0.90	0.57	0.8 (0.693–0.908)
D—The model combined reciprocal plasma MIF level and donor terminal Scr	0.162	0.96	0.66	0.53	0.98	0.62	0.872 (0.795–0.949)

*Scr, serum creatinine; PPV, positive predictive value; NPV, negative predictive values; AUC, area under curve; CI, confidence interval*.

### MIF Is Related to Graft Function 1-Month Post-transplant

**Figure 4:**
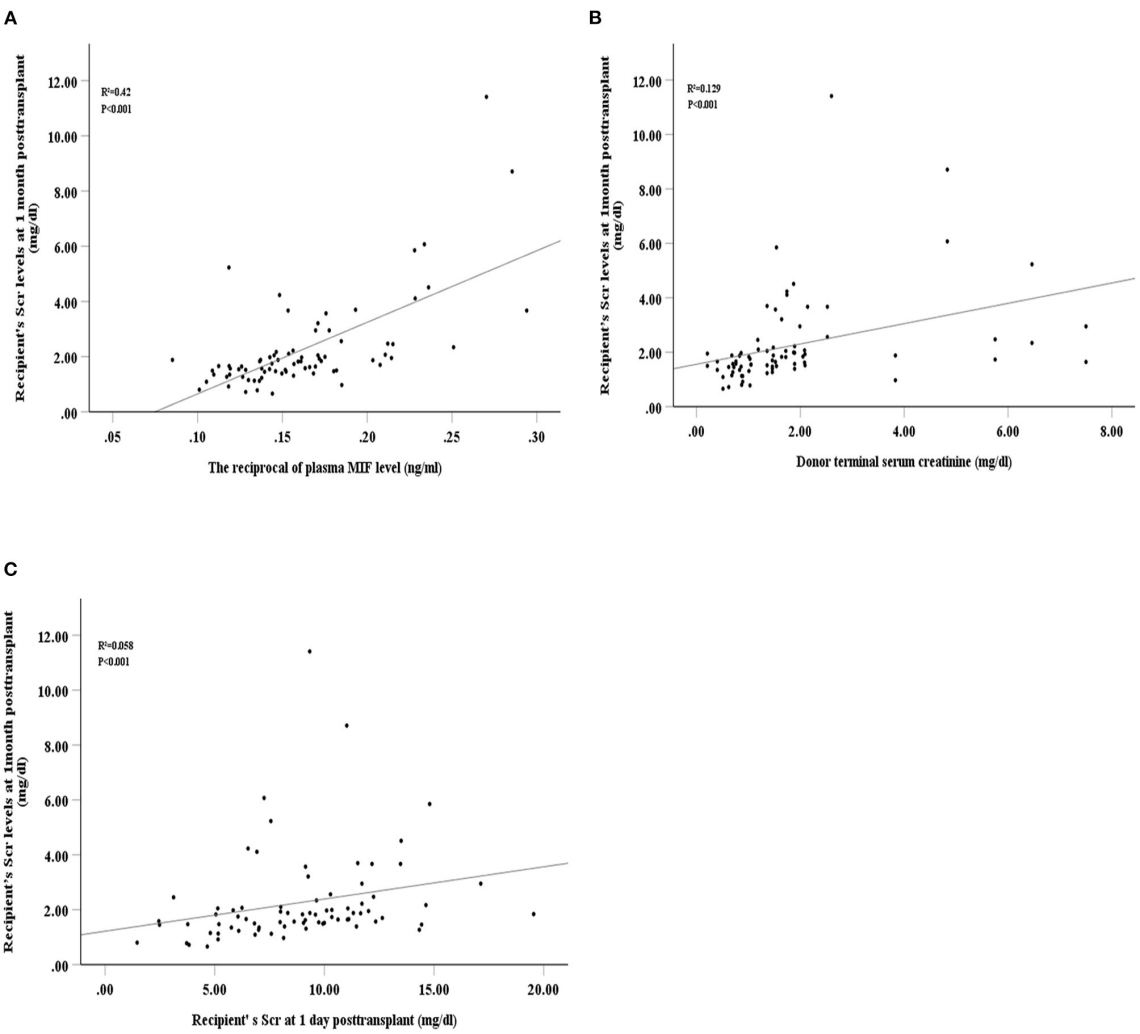
Spearman's correlation between recipient's serum creatinine at 1-month post-transplant and intraoperative plasma macrophage migration inhibitory factor (MIF) level **(A)**, donor terminal serum creatinine **(B)**, and recipient's serum creatinine at 1-day post-transplant **(C)**.

The reciprocal plasma MIF, donor terminal Scr, and recipient Scr at 1-day post-transplant were positively related with recipient Scr at 1-month post-transplant (*R*^2^ = 0.42, *P* < 0.001; *R*^2^ = 0.129, *P* < 0.001; *R*^2^ = 0.058, *P* < 0.001, respectively, Figures 4A–C). Univariate analyses showed that recipient Scr at 1-day post-transplant, plasma MIF, female donors, cause of donor death, donor terminal Scr and kidney cold ischaemic time were significantly related to recipient Scr 1-month post-transplant (β = 2.142, 95%CI 0.008–0.226, *P* = 0.035; β = −5.292, 95%CI −0.773 to −0.35, *P* = 0.001; β = −2.47, 95%CI −1.791 to −0.192, *P* = 0.016; β = −3.246, 95%CI −1.713 to −0.410, *P* = 0.002; β = 3.334, 95%CI 0.15–0.596, *P* = 0.001; β = 2.926, 95%CI 0.113–0.598, *P* = 0.005, respectively). Multivariate linear regression analysis revealed that kidney cold ischaemic time, donor cause of death, and plasma MIF were significantly associated with recipient Scr at 1-month post-transplant (β = 2.213, 95%CI 0.024–0.46, *P* = 0.03; β = −2.08, 95%CI −1.252 to −0.027, *P* = 0.041; β = −3.706, 95%CI −0.646 to −0.194, *P* = 0.001, respectively; Table 4).

**Table 4:** Univariate and multivariate linear regression analyses for predicting 1-month graft function.

	**Univariate**			**Multivariate**		
	**βcoefficient**	**95%CI**	***P-*value**	**βcoefficient**	**95%CI**	***P-*value**
Donor age (years)	0.824	−0.015 to 0.037	0.413			
Donor sex (women)	−2.47	−1.791 to −0.192	0.016			
Donor BMI (kg/m^2^)	1.895	−0.005 to 0.205	0.062			
Donor cause of death	−3.246	−1.713 to −0.410	0.002	−2.08	−1.252 to −0.027	0.041
Donor terminal Scr (mg/dL)	3.334	0.15 to 0.596	0.001			
Cold ischaemic time (h)	2.926	0.113 to 0.598	0.005	2.213	0.024 to 0.46	0.03
Recipient age (years)	0.387	−0.03 to 0.044	0.7			
Recipient sex (female)	−0.671	−1.058 to 0.525	0.504			
Duration of dialysis before transplantation (mo)	−0.38	−0.013 to 0.009	0.705			
HLA mismatch	−0.74	−0.583 to 0.267	0.462			
PRA	0.592	−0.78 to 1.44	0.556			
Recipient's Scr at 1-day post-transplant (mg/dL)	2.142	0.008 to 0.226	0.035			
Plasma MIF level (ng/mL)	−5.292	−0.773 to −0.35	0.001	−3.706	−0.646 to −0.194	0.001

*Multivariate linear regression analysis was executed using a stepwise variable selection procedure*.

*HLA, human leukocyte antigen; PRA, panel reactive antibody; Scr, serum creatinine; MIF, Macrophage migration inhibitory factor; BMI, body mass index; CI, confidence interval*.

## Discussion

Our study investigated the role of perioperative MIF release in predicting delayed graft function after KT and illustrated that the intraoperative plasma MIF was significantly lower in patients who developed DGF vs. patients with non-DGF. Moreover, the diagnostic value of intraoperative plasma MIF for predicting DGF was superior to that of the recipient's Scr at 1-day post-transplant. In addition, the combination of plasma MIF and donor terminal Scr has a high diagnostic value for predicting DGF. This may provide transplant physicians with an early, non-invasive, and accurate means of predicting graft function following KT.

MIF is a pleiotropic cytokine and its functions in AKI are not well understood. Previous studies have shown that MIF is a proinflammatory mediator of the innate immune system, and increased urinary MIF was related to the severity of kidney injury in glomerulonephritis or pyelonephritis (26, 27). Similarly, during sepsis or liver transplantation, increased MIF levels appear to aggravate renal damage (22, 28). However, recent studies have indicated that patients undergoing cardiac surgery with high circulating MIF had a significantly lower risk of developing AKI (21, 29). Furthermore, they demonstrated that MIF-deficient mice exhibited increased tubular cell damage and increased apoptotic tubular cells, and administering recombinant MIF ameliorated renal tubular injury and apoptosis in AKI mouse model (21). These findings suggested that in AKI induced by IRI, the protective effect of MIF on cell death may be more effective, surpassing the potential pro-inflammatory and chemokine-like effects of MIF. In addition, studies showed that MIF could bind to various receptors, including CXCR2, CXCR4, and CD74. The response to stress will be diverse when interacting with different receptors on different cells at different times. In our study, plasma MIF levels were elevated during the intraoperative period, and high MIF levels were associated with better graft recovery.

Firstly, our study showed that plasma MIF might be an independent protective parameter of DGF. We demonstrated that circulating MIF was increased during intraoperative and postoperative transplantation. MIF is rapidly released into the circulation from the affected cell pool under ischaemia, oxidative stress, inflammation or glucocorticoid, and the kidneys are protected by raised MIF (12, 13, 21, 23, 30). Several clinical studies have shown that MIF is elevated during and after liver transplantation or cardiac surgery (20–22), in accordance with our study. We believe that the source of intraoperative elevated MIF may be composed of two components. First, administering glucocorticoids before kidney reperfusion and surgical stimulation may induce MIF release in innate immune cells for recipients (23). Second, kidney ischaemia during organ procurement may stimulate MIF synthesis of tubular epithelial cells in donor and the reperfusion of graft may increase MIF release in epithelial cells (18). We further analyzed the difference in intraoperative and postoperative plasma MIF levels in 30 patients with different allograft function status and found that MIF was significantly higher in non-DGF patients than in DGF patients. Moreover, our study found that the difference was more remarkable during the intraoperative period. These results suggest that intraoperative MIF may be a potential protective factor for DGF. Next, we assessed the difference in intraoperative MIF levels in 77 patients with different allograft function statuses. Our results revealed that intraoperative MIF was significantly different between the IGF, SGF, and DGF groups, suggesting that it distinguishes between a more subtle allograft recovery pattern. Studies show that MIF has anti-inflammatory and anti-fibrotic effects in the kidney (30), and can alleviate IRI through cell protection and antioxidant mechanisms (18–21). We assumed that elevated MIF during transplantation protects the graft by alleviating the refusion injury of the graft. Our research results also showed that 78% of recipients had postoperative IGF when their plasma MIF level was >7.26 during the surgery. In addition, we speculated that MIF might be an endogenous protective factor in remote ischaemic preconditioning due to release from the ischaemic hypoxia issue and protection in remote organs, such as the heart and kidney. Consistent with our hypothesis, recent studies have reported that MIF was significantly elevated after remote ischaemic conditioning was carried out and alleviated cardiac IRI. However, the protective effect of MIF on the heart could be blocked by genetic or pharmacological blocking of MIF (31, 32). Most IRI damage begins in the reperfusion process (33), and the damage begins soon after transplanted kidney blood flow is reopened during KT. We deemed that circulatory MIF levels within 5 min of reperfusion reached a high level and were more likely to reflect the real protective effect of the graft than circulating MIF levels over a longer period after reperfusion.

Secondly, we found that plasma MIF yield a better predicting power compared with recipient Scr for DGF and improve the predictive power of donor terminal creatinine for DGF. DGF is caused by IRI and multiple contributing factors, including the characteristics of the donor and recipient (34). Early identification and stratification of high-risk patients with DGF may advance postoperative management and potentially improve short- and long-term outcomes of recipients undergoing transplantation (35). The diagnosis of DGF is still dependent on Scr and urine volume in recipient, which requires an understanding of both previous levels and changes, and is influenced by diuretics, which usually requires several days to confirm (36). The delay in diagnosis has greatly hindered clinical efforts to prevent and treat DGF. Our study confirmed that the predictive value of intraoperative plasma MIF and donor terminal Scr for DGF is better than that of recipient Scr. Furthermore, the model including plasma MIF and donor terminal Scr yield a higher predictive value for DGF than any of the two. Compared with postoperative Scr in the recipient, which is the most commonly used clinical indicator of kidney damage (37), we believe that the model can help transplant physicians recognize DGF early and make early treatment plans, such as optimizing fluid balance, timely appropriate dialysis, adjusting the dose of immunosuppressive agents, and avoiding the use of other nephrotoxic drugs (38, 39). Among the recently used AKI markers, kidney injury molecule-1 in tissues could not predict DGF (40), urinary neutrophil gelatinase-associated lipocalin (NGAL) and interleukin-18 have limited predictive value for DGF. Moreover, urine is not produced in most patients with DGF (39, 41). The value of these biomarkers in predicting DGF is limited. However, because of our results based a prospective cohort study with a small size and without validation cohort, these results should be interpreted with cautions. In order to study the exact role of MIF in protecting kidney and to validate the utility of plasma MIF levels as a biomarker for patients DGF prediction, further basic or clinical studies should be carried out.

Finally, we found that the reciprocal plasma MIF level during the intraoperative period was positively correlated with Scr at 1-month post-transplant, indicating that a high reciprocal plasma MIF was closely correlated with worse short-term renal graft function. Moreover, compared with the Scr levels of recipients and donors, intraoperative plasma MIF levels are more closely related to renal allograft function in the first month after transplantation. Studies have shown that urinary NGAL is correlated with renal allograft function at 3-weeks post-operation. However, it was not associated with long-term allograft function (39). Our follow-up time was short, and consequently, we were unable to assess the correlation between plasma MIF and long-term allograft function.

In conclusion, we believe that plasma MIF has a better predictive performance for DGF, which can help transplant physicians to make a preliminary judgement in the early postoperative period and provide individualized treatment plans for patients.

## Data Availability Statement

The original contributions presented in the study are included in the article/Supplementary Material, further inquiries can be directed to the corresponding author/s.

## Ethics Statement

The studies involving human participants were reviewed and approved by the Ethics Committee of the Third Affiliated Hospital of Sun Yat-sen University. The patients/participants provided their written informed consent to participate in this study.

## Author Contributions

QiqS and YY were responsible for study design, analysis and interpretation of data, and preparation and revision of the article. FH and YY performed the analysis. MM, HZ, and ZY performed the laboratory assays. QipS, ZH, ZL, and TL performed data collection. The article was edited by HL, LH, and NN. All authors have approved the manuscript for submission.

## Conflict of Interest

The authors declare that the research was conducted in the absence of any commercial or financial relationships that could be construed as a potential conflict of interest.

## Publisher's Note

All claims expressed in this article are solely those of the authors and do not necessarily represent those of their affiliated organizations, or those of the publisher, the editors and the reviewers. Any product that may be evaluated in this article, or claim that may be made by its manufacturer, is not guaranteed or endorsed by the publisher.

## Supplementary Material

Supplementary Figure 1 Distributions of plasma macrophage migration inhibitory factor (MIF) from the recipient at preoperative, intraoperative, and postoperative periods. Contents of MIF in non-delayed graft function (non-DGF; red) and delayed graft function (DGF; blue) groups.

Supplementary Figure 2 Variation of MIF from recipient during the perioperative period.

Supplementary Table 1 Summary of characteristics in recipient and donor, stratified by recipients's allograft function.

## Abbreviations

ATG: rabbit anti-human thymocyte immunoglobulin
AKI: acute kidney injury
BMI: body mass index
CNI: calcineurin inhibitor
CI: confidence interval
CRR: creatinine reduction ratio
DGF: delayed graft function
ESRD: end-stage renal disease
GN: glomerulonephritis
GCs: glucocorticoids
HD: hemodialysis
HLA: human leukocyte antigen
IGF: immediate graft function
IRI: ischaemia reperfusion injury
KT: kidney transplantation
MIF: macrophage migration inhibitory factor
NGAL: neutrophil gelatinase-associated lipocalin
OR: odds ratio
PD: peritoneal dialysis
PRA: panel reactive antibody
ROC: receiver operating characteristic
SGF: slowed graft function
Scr: serum creatinine
WD: without dialysis.
