# Supplementary figures and images for: Plasma Macrophage Migration Inhibitory Factor Predicts Graft Function Following Kidney Transplantation: A Prospective Cohort Study
Source: Front Med (Lausanne). 2021 Sep 1;8:708316. doi: 10.3389/fmed.2021.708316 (PMC8440878; doi:10.3389/fmed.2021.708316)

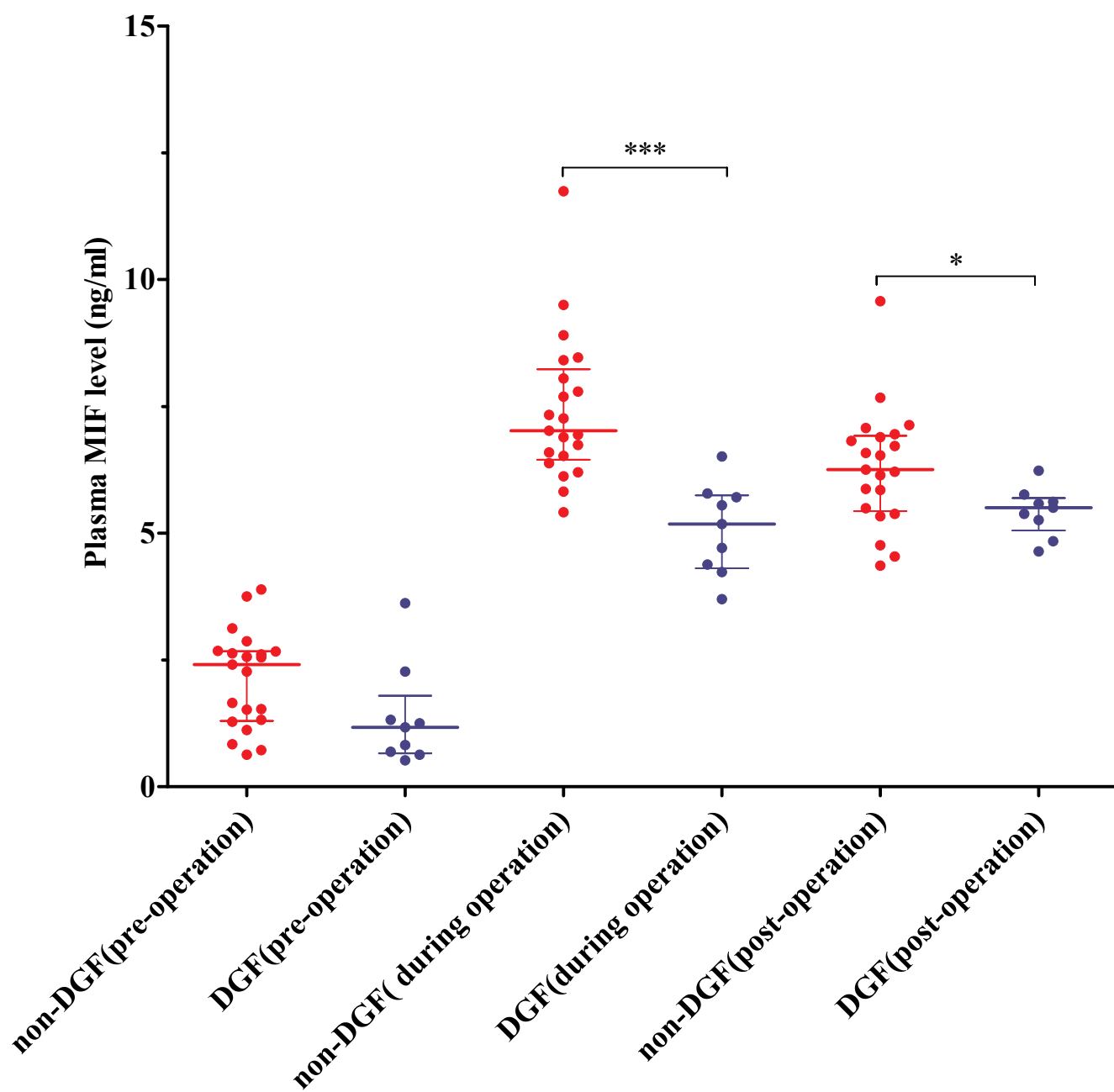

Supplement: Supplementary Figure 1 — Distributions of plasma macrophage migration inhibitory factor (MIF) from the recipient at preoperative, intraoperative, and postoperative periods. Contents of MIF in non-delayed graft function (non-DGF; red) and delayed graft function (DGF; blue) groups. [file Data_Sheet_1.PDF]

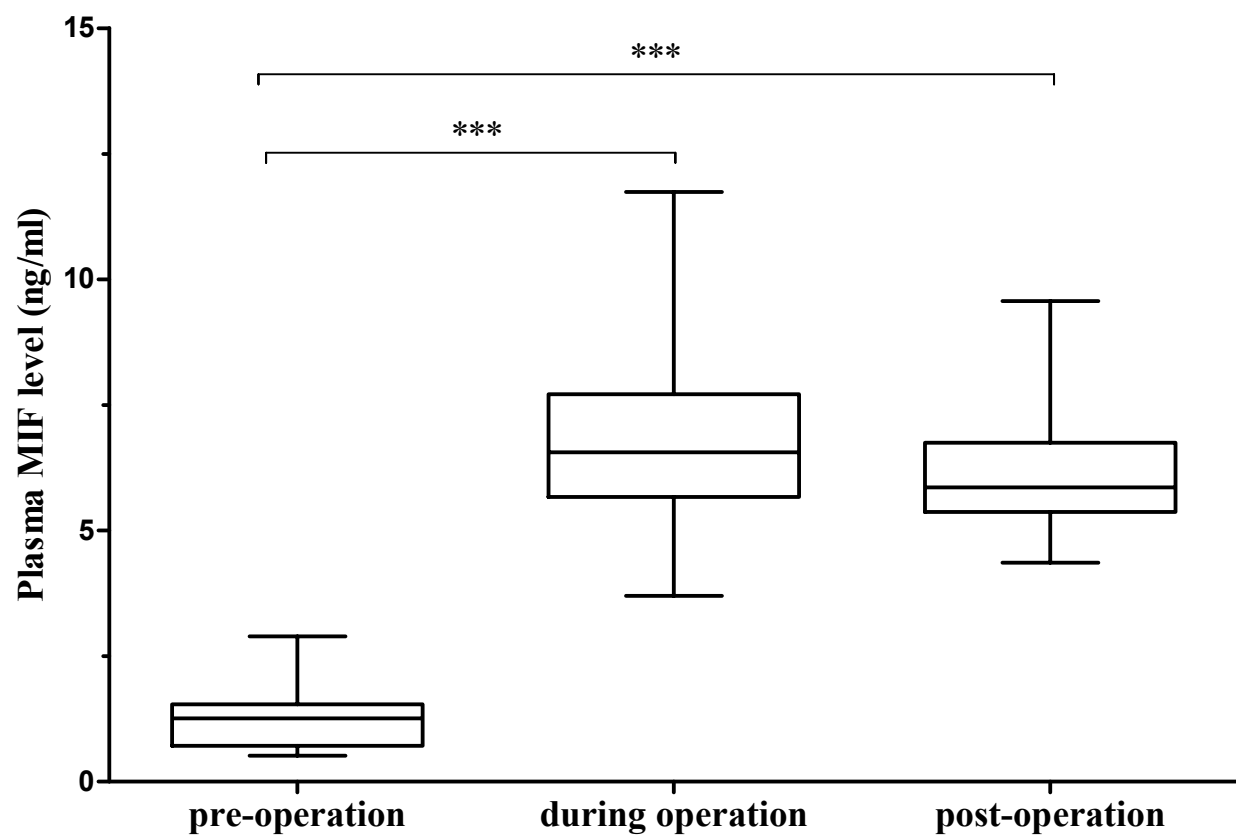

Supplement: Supplementary Figure 2 — Variation of MIF from recipient during the perioperative period. [file Data_Sheet_2.PDF]
