# Supplementary material for: Plasma Macrophage Migration Inhibitory Factor Predicts Graft Function Following Kidney Transplantation: A Prospective Cohort Study
Source: Front Med (Lausanne). 2021 Sep 1;8:708316. doi: 10.3389/fmed.2021.708316 (PMC8440878; doi:10.3389/fmed.2021.708316)
Supplement: Supplementary Table 1 — Summary of characteristics in recipient and donor, stratified by recipients's allograft function. [file Data_Sheet_3.PDF]

Supplement Table 1

Summary of characteristics in recipient and donor, stratified by recipients' s allograft function

Characteristic		All(n=30)	Non-DGF(n=21)	DGF(n=9)	*P* value
**Recipients**					
Age, years		45.5[35.8,50.5]	42[34.5,51]	49[40.5,53.5]	0.454
Male sex		17(57)	10(48)	7(78)	0.127
Cause of ESRD					0.231
	GN	8(27)	5(24)	3(33)	
	Hypertension	1(3)	0()	1(11)	
	Others	21(70)	16(76)	5(56)	
Mode of dialysis					0.291
	HD	26(87)	19(90)	7(78)	
	PD	3(10)	1(5)	2(22)	
	WD	1(3)	1(5)	0(0)	
Dialysis duration, mo		9.5[3,19.3]	10[3,23.5]	6[3.5,16]	0.821
Cold ischaemic time, h		4[3.5,5.3]	4[3.5,5]	5[4,6.5]	0.017
Number of HLA mismatches		5[4,5]	5[4,5.5]	5[4,5]	0.768
Panel reactive antibody					0.593
	0%	25(83)	17(81)	8(89)	
	1%-10%	5(17)	4(19)	1(11)	
Induction regimen					0.398
	ATG	20(67)	15(71)	5(56)	
	Basiliximab	10(33)	6(29)	4(44)	
CNI	Tacrolimus	30	21	9	
Steroid		30	21	9	
Renal functions at post-transplant (mg/dL)					
	SCr, at 1 day	8.17[6,01,10.34]	7.98[5.46,10.05]	10.34[7.47,12.87]	0.033
	SCr, at 1 week	3.06[1.5,5.92]	1.84[1.19,3.37]	6.64[4.75,8.56]	<0.001
	SCr, at 1 month	1.79[1.44,2.49]	1.55[1.19,1.95]	2.47[1.92,4.31]	0.004
Variation of Scr after transplantation (mg/dL)					
Absolute decrease	0 h to 1 day	1.50[-0.65,3.07]	1.91[-0.2,3.22]	-0.04[-0.93,1.39]	0.06
Relative decrease	(0 h to 1 day)/1 day	0.12[-0.08,0.34]	0.19[-0.01,0.34]	-0.01[-0.07,0.15]	0.013
MIF levels in pre-operation (ng/mL)		0.71[1.26,1.54]	1.28[0.78,1.54]	1.17[0.66,1.79]	0.428
MIF levels during operation (ng/mL)		6.56[5.67,7.72]	7.02[6.45,8.23]	5.18[4.31,5.75]	<0.001

MIF levels in post-operation (ng/mL)	5.86[5.36,6.75]	6.25[5.43,6.92]	5.5[5.05,5.69]	0.028
**Donors**				
Age, years	51[42,54]	44[40.5,52.5]	53[49.5,54.5]	0.07
Male sex	21(70)	15(48)	6(67)	0.749
BMI (kg/m^2)	23.7[20.1,26.1]	23.9[20.1,27.1]	23.1[21,24.4]	0.563
Hypertension	14(47)	10(48)	4(44)	0.873
Cause of death				0.984
Head trauma	3(10)	2(10)	1(11)	
Stroke	13(43)	9(43)	4(44)	
Other	14(47)	10(48)	4(44)	
Admission SCr, mg/dl	1.05[0.84,1.68]	1.04[0.71,1.3]	1.62[1.02,2.24]	0.048
Terminal SCr, mg/dl	1.36[0.85,2.06]	1.05[0.76,1.92]	1.87[1.28,2.33]	0.054
Use of any vasoactive drugs	26(87)	18(86)	8(89)	0.815
No. of any vasoactive drugs	2[2,3.8]	2[2,3]	2[2,7.5]	0.509
No. of kidneys transplanted 2	30	21	9	

According to Shapiro-Wilk test, if *P* > 0.05 for continuous variables, the data are presented as mean±SD; otherwise, the data are presented as median[P25,P75]; categorical variables are expressed by total

numbers and percentages.

ANOVA or Kruskal–Wallis tests were used to compare continuous variables and χ2 test and Fisher's exact test were used to compare categorical variables.

DGF, delayed graft function; ESRD, end-stage renal disease; GN, glomerulonephritis; HD, hemodialysis; PD, peritoneal dialysis; WD, without dialysis; HLA, human leukocyte antigen; ATG, Rabbit Anti-Human Thymocyte Immunoglobulin; CNI, calcineurin inhibitor; Scr, serum creatinine; MIF, Macrophage migration inhibitory factor; BMI, body mass index; AKI, acute kidney injury; ANOVA, analysis of variance.
